# Dynorphin-Dependent Reduction of Excitability and Attenuation of Inhibitory Afferents of NPS Neurons in the Pericoerulear Region of Mice

**DOI:** 10.3389/fncel.2016.00061

**Published:** 2016-03-11

**Authors:** Kay Jüngling, Peter Blaesse, Lena Goedecke, Hans-Christian Pape

**Affiliations:** Institute of Physiology I, University of MünsterMünster, Germany

**Keywords:** neuropeptide S, kappa opioid receptor, anxiety, stress, synaptic transmission

## Abstract

The Neuropeptide S system, consisting of the 20-amino acid peptide neuropeptide S (NPS) and its G-protein coupled receptor (NPSR), modulates arousal, wakefulness, anxiety, and fear-extinction in mice. In addition, recent evidence indicates that the NPS system attenuates stress-dependent impairment of fear extinction, and that NPS-expressing neurons in close proximity to the locus coeruleus region (LC; pericoerulear, periLC) are activated by stress. Furthermore, periLC NPS neurons receive afferents from neurons of the centrolateral nucleus of the amygdala (CeL), of which a substantial population expresses the kappa opioid receptor (KOR) ligand precursor prodynorphin. This study aims to identify the effect of the dynorphinergic system on NPS neurons in the periLC via pre- and postsynaptic mechanisms. Using electrophysiological recordings in mouse brain slices, we provide evidence that NPS neurons in the periLC region are directly inhibited by dynorphin A (DynA) via activation of κ-opioid receptor 1 (KOR1) and a subsequent increase of potassium conductances. Thus, the dynorphinergic system is suited to inactivate NPS neurons in the periLC. In addition to this direct, somatic effect, DynA reduces the efficacy of GABAergic synapses on NPS neurons via KOR1 and KOR2. In conclusion, the present study provides evidence for the interaction of the NPS and the kappa opioid system in the periLC. Therefore, the endogenous opioid dynorphin is suited to inhibit NPS neurons with a subsequent decrease in NPS release in putative target regions leading to a variety of physiological consequences such as increased anxiety or vulnerability to stress exposure.

## Introduction

The neuropeptide S (NPS) system, consisting of the 20-amino acid peptide NPS and its G-protein coupled receptor (NPSR), has been shown to be involved in processes of anxiety, fear-extinction, and fear memory consolidation (Xu et al., 2004; Okamura and Reinscheid, 2007; Jüngling et al., 2008; Okamura et al., 2011). Recent evidence suggests that the NPS system is involved in stress coping and that stressful events increase the release of NPS in the amygdala of rodents (Ebner et al., 2011; Chauveau et al., 2012). In line with this, immobilization stress induces an up-regulation of c-fos in NPS-expressing neurons, indicating an activation of these neurons during periods of stress (Liu et al., 2011; Jüngling et al., 2012). The NPS neurons express the corticotropin-releasing factor receptor 1 (CRF1) and depolarize following CRF1 activation by CRF or stressin I (Liu et al., 2011; Jüngling et al., 2012).

Stressful conditions additionally promote the activation of endogenous opioid systems (for review, Knoll and Carlezon, 2010) and the release of dynorphin A (DynA), which in turn activates κ-opioid receptors (KOR; Chavkin et al., 1982). Activation of the dynorphin system induces analgesia, but also aversive and prodepressive-like behavior in rodents (Mague et al., 2003; Carlezon et al., 2006; Bruchas et al., 2007; Land et al., 2008, 2009; Knoll and Carlezon, 2010). The activation of KORs has been shown to modulate the excitability of neurons and directly influence transmitter release by presynaptic mechanisms. Depending on the cell type and brain region, dynorphin can either increase or decrease the activity of neurons. An increase in activity can be based on e.g., the enhancement of a hyperpolarization-activated current (I_h_; Pan, 2003) or on the modulation of voltage-dependent potassium currents (McDermott and Schrader, 2011). In contrast, a decrease in neuronal activity can be mediated by an enhancement of potassium conductances, most likely inwardly rectifying potassium conductances (Grudt and Williams, 1993, 1995; Ma et al., 1995). In rodents, agonists of KORs attenuate the release of various neurotransmitters and neuropeptides on the norepinephrine neurons of the locus coeruleus (LC) by presynaptic inhibition (Kreibich et al., 2008). Neurons of the LC receive afferents from the central amygdala (Dimitrov et al., 2013) and these afferents can contain CRF and/or dynorphin (Reyes et al., 2008, 2011). NPS-expressing neurons in the periLC region have recently been shown to receive central amygdalar afferents originating from dynorphin- and somatostatin-expressing neurons (Jüngling et al., 2015). In addition, NPS neurons are inactivated by application of DynA or somatostatin, indicating a negative control of NPS-expressing neurons by these peptide systems (Jüngling et al., 2015).

Based on the findings that the NPS system is involved in stress responses and that NPS neurons in the periLC respond to CRF and DynA, two key players of stress responses, we used *in vitro* slice electrophysiology to analyze the effects of DynA on NPS neurons in more detail and to identify the underlying synaptic mechanisms. In addition to the direct inhibitory effect of DynA on NPS neurons, we analyzed its role in the regulation of synaptic efficacy of GABAergic afferents projecting onto NPS neurons.

## Materials and Methods

### Animals

Heterozygous transgenic mice expressing the enhanced green fluorescent protein (EGFP) under the control of the NPS promotor (transgenic NPS-EGFP mouse line E16; Liu et al., 2011) were bred with C57Bl/6 mice and offspring was genotyped by PCR as described previously (Liu et al., 2011). Mice were kept in a temperature (21°C) and humidity-controlled (50–60% relative humidity) animal facility with access to food and water ad libitum and a 12:12 h light-dark cycle with lights on at 6:00 AM. All animal experiments were carried out in accordance with national regulations on animal experimentation (European Committees Council Directive 86/609/EEC; National Research Council of the National Academies) and protocols were approved by the local authorities (Bezirksregierung Münster, AZ 50.0835.1.0, G 53/2005) and “Landesamt für Natur, Umwelt und Verbraucherschutz Nordrhein-Westfalen” (reference number: 8.87-51.05.20.10.218).

### Electrophysiology

Six to ten weeks old NPS-EGFP mice of either sex were anesthetized with isoflurane (Forene, 1-Chloro-2,2, 2-trifluoroethyl-difluoromethylether; 2.5%) and killed by decapitation. Horizontal slices (300 μm thick) containing the LC were prepared. Whole-cell patch-clamp recordings (in voltage- or current-clamp mode) were performed as described previously (Jüngling et al., 2008). We used patch pipettes made of borosilicate glass (GC150T-10, Harvard Apparatus, Edenbridge), pulled on a vertical puller (PA-10, E.S.F. Electronic, Germany). The intracellular solution used to analyze the intrinsic properties of NPS-EGFP neurons contained [in mM]: NaCl 10, K-gluconate 105, K_3_-citrate 20, HEPES 10, BAPTA 3, MgCl_2_ 1, MgATP 3, and NaGTP 0.5. The pH was adjusted to 7.25. Artificial cerebrospinal fluid (aCSF) was used as extracellular solution and contained [in mM]: NaCl 120, KCl 2.5, NaH_2_PO_4_ 1.25, MgSO_4_ 2, CaCl_2_ 2, and glucose 20. The pH was adjusted to 7.3 by gassing with carbogen (95% O_2_, 5% CO_2_). The liquid junction potential was corrected for (10 mV). The pipette resistance was between 2.2 and 2.8 MΩ using the intracellular solution listed above. The series resistance RS was between 5–15 MΩ and recordings with higher RS were rejected from analysis. All experiments were performed at 30–32°C.

Active and passive membrane properties were assessed during whole-cell current-clamp recordings at a membrane potential of −60 mV. Hyper- and depolarizing currents were injected for 500 ms (injected currents from −80 to +140 pA; ΔI +10 pA). Active membrane properties were analyzed during depolarizing current injections of +80 to +120 pA. The input resistance of the recorded neurons was calculated by R_input_ − ΔV/I. ΔV was determined under steady-state conditions at the end of an injected hyperpolarizing current pulse (I = −50 pA). The membrane time constant τ was obtained by a monoexponential fit of the membrane potential shift. The resting membrane potential was measured immediately after establishing the whole-cell configuration. The after-hyperpolarizing potential (AHP) was determined after the first action potential (AP). The slow AHP (sAHP) was defined as hyperpolarizing voltage deflection following a 500 ms current injection of +80 pA. The AP half-width was extracted from the first AP. The time-to-first-AP was calculated as time from the beginning of the depolarizing current injection (+80 pA) to the onset of the first AP. The instantaneous frequency was calculated between the first two APs occurring in response to a depolarizing current injection of +80 pA.

Electrophysiological data were acquired with an EPC10-double amplifier (HEKA, Germany) at a sampling rate of 10 kHz and analyzed offline with Clampfit10 software (Molecular Devices Corporation, Sunnyvale, CA, USA).

### Drug Testing

To analyze the effect of opioid receptor agonists, NPS-EGFP neurons were recorded either in the current-clamp mode at a membrane potential around −65 mV or in the voltage-clamp mode at −65 mV. Drugs were bath-applied for 5 min at a perfusion speed of ~3.5 ml/min. In the current-clamp mode, the input resistance was monitored by injecting hyperpolarizing currents of −50 pA. During maximal drug effect, the membrane potential was manually set to baseline values by adjusting a DC offset to exclude changes of the input resistance induced by voltage-dependent conductances. Gabazine (GBZ; 25 μm) or picrotoxin (100 μm), CGP55845 (10 μm), D-(-)-2-Amino-5-phosphonopentanoic acid (AP5, 50 μm), and 6,7-Dinitroquinoxaline-2,3-dione (DNQX, 10 μm) were added to the bathing solution as required to block GABAergic and glutamatergic postsynaptic currents, respectively. In some experiments, tetrodotoxin (TTX, 0.5–1 μm) was added to decrease network activity (toxins were purchased from Biozol Diagnostica, Germany).

The KOR agonist DynA, the μ-opioid receptor (MOR) agonist [D-Ala2, N-MePhe4, Gly-ol]-enkephalin (DAMGO), and the δ-opioid receptor (DOR) agonist SNC-80 were applied at a concentration of 500 nM (all from Tocris). The KOR antagonist GNTI (5^′^-Guanidinyl-17-(cyclopropylmethyl)-6,7-dehydro-4,5α-epoxy-3,14-dihydroxy-6,7-2^′^,3^′^-indolomorphinan dihydrochloride; Tocris) was applied prior to DynA at a concentration of 1.5 μm. The κ-opioid receptor 1 (KOR1) agonist U69,593((+)-(5α,7α,8β)-N-Methyl-N-[7-(1-pyrrolidinyl)-1-oxaspiro[4.5]dec-8-yl]-benzeneacetamide; Sigma Aldrich) and the KOR2 agonist GR 89696 fumarate (4-[(3,4-Dichlorophenyl)acetyl]-3-(1-pyrrolidinylmethyl)-1-piperazinecarboxylic acid methyl ester fumarate, Tocris) were bath-applied at a concentration of 250 nM. For co-application of DynA and CRF (Tocris), both drugs were used at a concentration of 250 nM. The CRF receptor 1 (CRF1) antagonist antalarmin hydrochloride (Tocris) was used at a concentration of 4 μm. Tertiapin Q (Tocris), an antagonist of ROMK1 and GIRK1/4 potassium channels, was bath-applied at a concentration of 200 nM. In some experiments, 0.5 μm BaCl_2_ was added to the aCSF. When using BaCl_2_, MgSO_4_ was replaced by 1.5 mM MgCl_2_ in the aCSF.

### Voltage-Clamp Ramps

To analyze the nature of substance-induced currents, depolarizing voltage-clamp ramps were performed (from −120 to −20 mV; 3 s) from a holding potential of −60 mV. The recordings were done in presence of 1 μm TTX to reduce network activity and 100 μm CdCl_2_ to reduce calcium currents during depolarizing ramps. Ramps were recorded during baseline conditions (Ramp_baseline_) and during maximal drug effect (Ramp_drug_). The resultant, substance-induced current was calculated by Ramp_drug_ − Ramp_baseline_ and plotted vs. the holding potential.

### Evoked Inhibitory Postsynaptic Currents (eIPSCs)

To evoke GABA_A_ receptor (GABA_A_R)-mediated postsynaptic currents in NPS neurons, a tungsten bipolar stimulation electrode was placed rostral to the NPS neurons in the periLC. NPS neurons were recorded in voltage-clamp mode at a holding potential of −65 mV using a KCl-based intracellular solution. Recordings were done in presence of AMPA, NMDA and GABA_B_ receptor antagonists. The latency was measured between the stimulation artifact and the onset of the postsynaptic responses. Responses with latencies <5 ms were accepted for analysis. Evoked inhibitory postsynaptic currents (eIPSCs) were considered as failures when it lacked typical IPSC kinetics and when the amplitude was smaller than two times the standard deviation of the mean baseline noise. Brief (50–100 μs) electrical pulses were delivered with an interstimulus interval of 20 s. The stimulation strength was set to allow failures in the majority of recordings during baseline stimulation.

### Miniature Inhibitory Postsynaptic Currents (mIPSCs)

The miniature IPSCs (mIPSCs) were recorded in the voltage-clamp mode at a holding potential of −65 mV using a high chloride intracellular solution, containing 105 mM KCl instead of K-gluconate. To block AP triggered release, 0.5 μm tetrodotoxin was added to the aCSF. DNQX, AP-5, and CGP55845 were added to the aCSF to antagonize AMPA-, NMDA-, and GABA_B_ receptor-mediated currents, respectively, and to record pharmacologically isolated GABA_A_R-mediated mIPSCs. Neurons were allowed to equilibrate for at least 5 min prior to recordings. Frequency, amplitudes and kinetics of mIPSCs, rise time_10–90%_ and decay time_90–10%_, were automatically analyzed during baseline conditions and in presence of DynA using Clampfit10.

### Statistics

Data are represented as box plots, in which the box represents the first (25%) and third (75%) quartiles, the band represents the median, and the whiskers represent the percentiles 5% and 95%. The square within the box represents the mean. All data sets were tested for statistically significant outliers using the Grubbs’ test (significance level *p* < 0.05). Within group comparisons were done by using a paired student’s *t*-test (significance level *p < 0.05; **p < 0.01). To analyze differences between different groups, one-way ANOVA followed by Bonferroni *post hoc* test or unpaired student’s *t*-test were used (significance level *p < 0.05; **p < 0.01).

## Results

**Figure 1:**
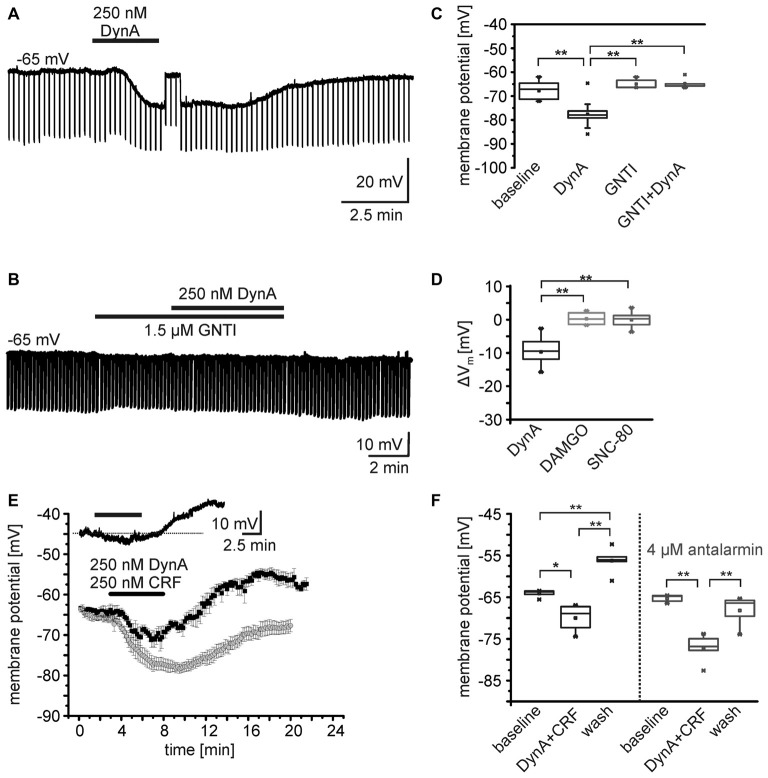
**Effects of opioid receptor agonists on the membrane potential of NPS neurons. (A,B)** Sample current-clamp recordings of NPS neurons in the periLC region. Hyperpolarizing current pulses (−50 pA; 500 ms) were applied to monitor the input resistance of the recorded neuron. While application of 250 nM DynA induced a transient hyperpolarization **(A)**, the kappa opioid receptor (KOR) antagonist GNTI hydrochloride prevented the DynA effect **(B)**. To monitor drug-induced changes in input resistance, the membrane potential was manually set to baseline values by adjusting a DC offset during maximal drug effect (transient jump in membrane potential seen in **A**). This allows distinguishing between direct drug-induced changes in input resistance and indirect changes induced by voltage-dependent conductances. **(C)** Quantification of the effect of DynA on the membrane potential in the absence (*n* = 14) or presence of GNTI hydrochloride (*n* = 5). **(D)** Quantification of the mean membrane potential shift following application of 250 nM DynA (*n* = 14), 500 nM DAMGO (*n* = 9) and 500 nM SNC-80 (*n* = 9). **(E)** Time course of the membrane potential changes in response to DynA/CRF co-application in absence (black) or presence (gray) of the corticotropin-releasing factor receptor 1 (CRF1) antagonist antalarmin. Inset shows a sample recording in current-clamp mode. **(F)** Quantification of the membrane potential changes in NPS neurons during DynA/CRF co-application (*n* = 5) and DynA/CRF co-application in presence of antalarmin (*n* = 5).

Gene expression profiling of isolated NPS neurons from the periLC of NPS-EGFP mice indicates the expression of KORs in these neurons (Liu et al., 2011). Furthermore, it has previously been shown that the neuropeptides DynA and somatostatin hyperpolarize NPS neurons in the periLC region (Jüngling et al., 2015). To reveal detailed information on the underlying cellular mechanisms and the specificity of the DynA application and the subsequent activation of KORs, EGFP-expressing NPS neurons (here referred to as NPS neurons) were recorded in the whole-cell current-clamp mode at a membrane potential set around −65 mV. In the presence of glutamate and GABA receptor antagonists, and 0.5 μm TTX, the application of 250 nM DynA induced a significant membrane hyperpolarization in all neurons tested (*n* = 14; Figures 1A,C). In agreement with previous results, the average membrane potential (V_m_) of NPS neurons during baseline conditions was at –67.9 ± 1.1 mV and shifted to –77.6 ± 1.4 mV in the presence of DynA (ΔV_m_ = –9.5 ± 1.0 mV; *p* = 6.806E-7; *t* = 9.42; DoF = 12; Figure 1D). The DynA-induced hyperpolarization was absent in the presence of the KOR antagonist GNTI (1.5 μm; *n* = 5; Figures 1B,C). The membrane potential was at –64.9 ± 1.0 mV in the presence of GNTI and at –64.8 ± 1.0 mV in the presence of GNTI and DynA (ΔV_m_ = 0.14 ± 0.68 mV). GNTI alone had no significant effect on the membrane potential of NPS neurons (One-Way analysis of variance [ANOVA]: *F*_(3,32)_ = 21.7; *post hoc* test: GNTI vs. GNTI/DynA: *p* = 1; baseline vs. DynA: *p* = 4.37E-6; DynA vs. GNTI/DynA: *p* = 6.065E-6; baseline vs. GNTI: *p* = 1; DynA vs. GNTI: *p* = 7.368E-6; Figures 1B,C). These data indicate that DynA hyperpolarizes NPS neurons in the periLC via activation of KORs.

In a next set of experiments, the specific MOR and DOR agonists DAMGO and SNC-80, respectively, were bath-applied during current-clamp recordings. Neither 500 nM DAMGO (V_m_ = –63.1 ± 1.3 mV vs. baseline V_m_ = –63.4 ± 1.4 mV; ΔV_m_ = −0.28 ± 0.61 mV, *n* = 9; *p* = 0.663; *t* = −0.453; DoF = 8), nor 500 nM SNC-80 (V_m_ = –63.7 ± 1.4 mV vs. baseline V_m_ = –63.7 ± 1.0 mV; ΔV_m_ = –0.06 ± 0.77 mV; *n* = 9; *p* = 0.944; *t* = 0.072; DoF = 8) changed the V_m_ of NPS neurons (Figure 1D). The ΔV_m_ induced by DynA was significantly different from the ΔV_m_ induced by DAMGO or SNC-80 (One-way ANOVA *F*_(2,28)_ = 43.3; *p* = 2.704E-9; *post hoc* test: DynA *vs*. DAMGO: *p* = 3.593E-8; DynA *vs*. SNC-80: *p* = 6.949E-8; DAMGO vs. SNC-80: *p* = 1; Figure 1D). These data indicate that NPS neurons at the periLC are responsive to KOR activation, but do not respond to MOR or DOR agonists.

As previously reported, NPS neurons in the periLC depolarize following activation of CRF1 (Jüngling et al., 2012). Since DynA and CRF can be coexpressed in LC afferents (Reyes et al., 2008; for review, see Van Bockstaele et al., 2010), we tested the effect of co-application of DynA and CRF (250 nM each) on the membrane potential of NPS neurons in the periLC. Under current-clamp conditions at a membrane potential of −65 mV (−64.1 ± 0.9 mV) in presence of 1 μm TTX, the co-application induced a hyperpolarization (−69.9 ± 3.3 mV) followed by a prominent depolarization (−56.2 ± 3.3 mV) upon wash (One-Way ANOVA: *F*_(2,12)_ = 33.06; *p* = 1.313E-5; *post hoc* test: baseline vs. DynA/CRF: *p* = 0.014; baseline vs. wash: *p* = 0.002; DynA/CRF vs. wash: *p* = 9.893E-6; *n* = 5; Figures 1E,F). Co-application of DynA and CRF in the presence of the CRF1 antagonist antalarmin (4 μm) induced a more prominent and longer lasting hyperpolarization and abolished the depolarization during wash (One-Way ANOVA: *F*_(2,12)_ = 22.7;*post hoc* test: baseline vs. DynA/CRF: *p* = 9.258E-5; baseline vs. wash: *p* = 0.416; DynA/CRF vs. wash: 0.001; *n* = 5; Figures 1E,F). The DynA/CRF-induced hyperpolarization was significantly larger in presence of antalarmin (ΔV_m_: −11.9 ± 1.7 mV) compared to recordings without antalarmin (ΔV_m_: −5.9 ± 1.2 mV; unpaired *t*-test: *p* = 0.022; *t* = −2.84; DoF = 8). These findings indicate that the dynorphin and the CRF system interact at the level of single NPS neurons and induce a biphasic change in membrane potential in these cells.

**Figure 2:**
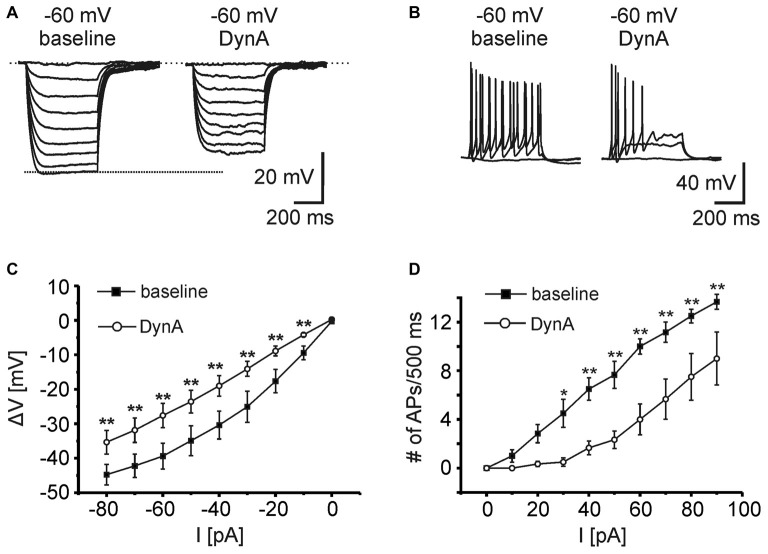
**KOR activation by DynA reduces the excitability of NPS neurons. (A)** Passive membrane responses to hyperpolarizing current injections (−80 to 0 pA; ΔI +10 pA; 500 ms duration) in absence and presence of 250 nM DynA (*n* = 14). In presence of DynA, NPS neurons showed reduced voltage deflections in response to hyperpolarizing current injections. **(B)** Quantification of passive membrane responses to hyperpolarizing current injections. **(C)** Active membrane responses of NPS neurons to increasing depolarizing current injections (0–90 pA; ΔI +10 pA; 500 ms duration). Application of 250 nM DynA reduced the number of action potentials (APs) generated in response to the injected current. **(D)** Quantification of active membrane responses to depolarizing current injections.

In order to analyze the effect of DynA on active and passive membrane properties of NPS neurons, current-clamp recordings in the absence of TTX were done (Figures 2A,B). Hyper- and depolarizing current injections (500 ms duration; from −80 to +100 pA; ΔI +10 pA) were delivered during current-clamp recordings at a membrane potential of −60 mV under baseline conditions and in presence of 250 nM DynA (Figures 2A,B). In the presence of DynA, the shift of the membrane potential in response to hyperpolarizing currents was significantly less at all tested currents (Figures 2A,C). These data indicate that DynA induces a reduction in input resistance, which is in line with previous observations (Jüngling et al., 2015). Additionally, in the presence of DynA, NPS neurons generated fewer APs in response to depolarizing current injections, reaching significance for all tested current injections from +30 pA on (Two-Way ANOVA with repeated measurements: *F*_(9,252)_ = 10.04; there is a significant difference between baseline and DynA: *p* < 0.0001; levels of significance for the *post hoc* test are indicated in the Figure 2D). A summary of other active and passive membrane properties analyzed during baseline conditions and in presence of DynA is given in Table 1 (*n* = 14). In summary, DynA reduces the input resistance and excitability of NPS neurons.

**Table 1:** **Summary of passive and active membrane properties recorded in the current-clamp mode at a membrane potential of −60 mV during baseline and in presence of DynA (*n* = 14)**.

	Baseline		DynA	
	Mean	SEM	Mean	SEM	*p*-value
R_in_ [MΩ]	629	51	431	43	**0.01**
Tau [ms]	38.2	1.9	32.7	3.3	0.17
Threshold [mV]	−41.2	0.6	−38.9	1.0	*0.06*
AP amplitude [mV]	73.7	0.7	68.4	0.9	**0.03**
AHP [mV]	17.6	0.6	18.8	0.5	0.15
Half-width [ms]	1.2	0.1	1.1	0.1	0.31
slAHP [mV]	3.1	0.5	1.4	0.3	**0.01**
Amplitude_last_ [mV]	53.7	2.2	53.3	2.8	0.92
AHPlast [mV]	17.0	0.9	18.8	0.7	0.13
Inst. frequency [Hz]	34.8	1.7	28.7	2.5	*0.06*
Time to 1st AP [ms]	12.2	0.8	14.0	1.3	0.25

*Significant changes are marked in bold. Differences with a strong trend, but not reaching the *p* < 0.05 significance criteria are marked in italic*.

### Ionic Mechanisms Underlying DynA-Induced Hyperpolarization

**Figure 3:**
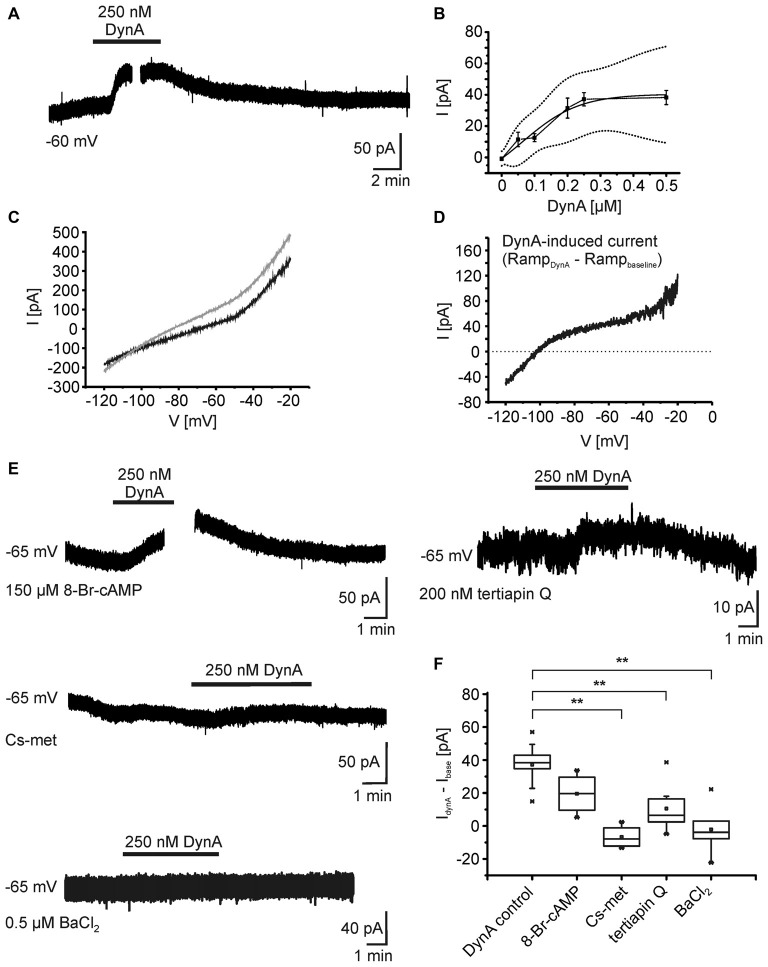
**Ionic mechanisms underlying the DynA-induced hyperpolarization. (A)** Example of NPS neuron recorded in the voltage-clamp mode at a holding potential of −60 mV. Application of 250 nM DynA induces a transient, outward directed current. The gap in the trace indicates the time point of a voltage-clamp ramp recording during DynA application. **(B)** Dose-response relationship of the DynA-induced outward current in response to 50 nM (*n* = 4), 100 nM (*n* = 3), 200 nM (*n* = 6), 250 nM (*n* = 9) and 500 nM (*n* = 8) DynA. Dotted lines represent the upper and lower confidence interval (95% level). **(C)** Sample voltage-clamp ramp recording (−120 mV to −20 mV, 3 s, 60 s inter-trial interval) during baseline (black) and in presence of DynA (gray). **(D)** Example of a DynA-induced current calculated from the voltage-clamp ramps shown in **(C)**. The mean DynA-induced current reversed at −106.5 ± 4.6 mV (*n* = 5). **(E)** Example recordings of the DynA-induced current with inclusion of either 8-Br-cAMP or Cs-met in the internal recording solution or in presence of either 200 nM tertiapin Q or 0.5 μm BaCl_2_. **(F)** Quantification of the DynA-induced current during control conditions (*n* = 9), in the presence of 8-Br-cAMP (*n* = 4), Cs-met-sulfonate (*n* = 4), tertiapin Q (*n* = 8) or BaCl_2_ (*n* = 6).

**Figure 4:**
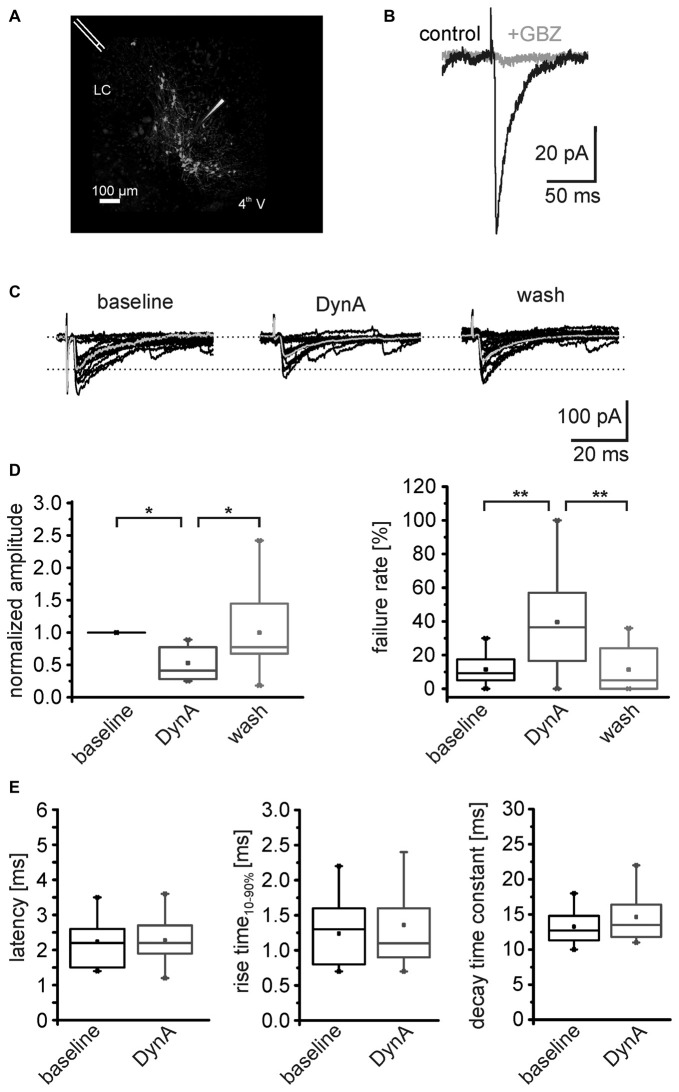
**DynA reduces the efficacy of GABAergic synapses on NPS neurons. (A)** Scheme of the stimulation electrode position and recording site in a horizontal slice preparation of a transgenic nps-enhanced green fluorescent protein (EGFP) mouse. NPS neurons are located close to the fourth ventricle (4th V) next to the locus coeruleus (LC). The stimulation electrode was placed rostrally to the recording site. **(B)** Evoked GABAergic postsynaptic responses (black) were sensitive to the GABA_A_receptor (GABA_A_R) antagonist gabazine (GBZ). **(C)** Examples of electrically evoked IPSCs (eIPSCs) during baseline, in presence of 250 nM DynA and after wash. The mean response is presented in gray. **(D)** Quantification of the mean normalized success amplitudes and failure rate of the eIPSCs (*n* = 11). **(E)** Quantification of the eIPSC latency, rise time_10–90%_ and decay time constant during baseline and in presence of DynA.

In order to reveal the ionic mechanisms underlying the hyperpolarization of NPS neurons following KOR activation, whole-cell voltage-clamp recordings were performed at a holding potential of −60 mV in presence of DNQX, AP-5, GBZ, CGP55845, and TTX (Figure 3A). In voltage-clamp mode, 250 nM DynA induced an outward current of 34.9 ± 4.7 pA (*n* = 5). The current amplitude was dose-dependent (50 nM: 11.5 ± 4.6 pA, *n* = 4; 100 nM: 12.6 ± 2.6 pA, *n* = 3; 200 nM: 31.5 ± 6.4 pA, *n* = 6; 250 nM: 37.2 ± 4.2 pA, *n* = 9; 500 nM: 38.3 ± 4.5 pA, *n* = 8; Figure 4B). The dose-response curve indicates that 250 nM DynA induced a near-maximal activation of KORs, since 500 nM DynA did not induce a larger current (Figure 3B).

To identify the DynA-induced current, a voltage-clamp ramp protocol (−120 to −20 mV, 3 s, 60 s inter-trial interval) was applied during baseline conditions and in the presence of DynA (Figure 3C). The DynA-induced current was calculated by subtraction of the currents during baseline conditions from those recorded in the presence of DynA (Figure 3D), and current-voltage (I-V) relations were constructed. DynA induced an outward current that reversed at −106.5 ± 4.6 mV (*n* = 5), a value close to the estimated potassium equilibrium potential for the present recording conditions (−109 mV). In addition, the DynA-induced current displayed slight inward rectification. To test a possible effect of DynA on adenylyl cyclase-dependent pathways, 150 μm 8-Br-cAMP, which clamps the intracellular cAMP level at a high level, was included in the recording pipette during voltage-clamp recordings. The inclusion of 8-Br-cAMP reduced the DynA-induced outward current in NPS neurons without reaching statistical significance (DynA-induced current in control: 37.2 ± 4.2 pA; *n* = 9; DynA-induced current in presence of 8-Br-cAMP: 19.6 ± 6.3; *n* = 4; Figure 3F), suggesting that the observed current is mediated in parts via a down-regulation of AC activity. Furthermore, a cesium-methanesulfonate based intracellular solution (Cs-met) abolished the DynA-induced current completely (DynA-induced current with Cs-met: −6.7 ± 3.5 pA; *n* = 4), providing further evidence for involvement of potassium conductances following KOR stimulation. Blocking ROMK1 and GIRK1/4 potassium channels by adding 200 nM tertiapin Q reduced the DynA-induced current to 10.6 ± 4.8 pA (*n* = 8; Figures 3E,F). The DynA-induced current was completely abolished in 0.5 μm BaCl_2_, which blocks a broad spectrum of inwardly rectifying potassium channels (DynA-induced current in BaCl_2_: −2.1 ± 5.9 pA, *n* = 6; Figures 3E,F; One-Way ANOVA: *F*_(4,26)_ = 12.67; *p* = 7.497E-6; *post hoc* test: DynA control *vs*. CsCl_2_: *p* = 5.283E-5; DynA control *vs*. tertiapin Q: *p* = 0.002; DynA control *vs*. BaCl_2_: *p* = 3.907E-5). These data provide evidence that the DynA-induced current is mediated by inwardly rectifying potassium channels and that the majority of these channels are tertiapin Q-sensitive GIRK1/4 channels.

### DynA Negatively Modulates GABAergic Transmission

In a next set of experiments, the AP-dependent release of GABA was investigated by analyzing electrically evoked IPSCs (eIPSCs) in NPS neurons. The stimulation electrode was positioned rostro-laterally to the NPS neuron cluster (Figure 4A). The eIPSCs appeared as inward currents and were sensitive to the GABA_A_R antagonist GBZ (*n* = 3; Figure 4B). The eIPSCs had a mean amplitude of –122 ± 31 pA with a failure rate of 11.2 ± 2.9% (*n* = 11). Application of 250 nM DynA reduced normalized mean eIPSC amplitudes to 0.53 ± 0.07. This effect was completely reversed upon wash out of DynA. At the end of the recording, the amplitude of eIPSCs recovered to 0.99 ± 0.18 of baseline (One-Way ANOVA: *F*_(2,30)_ = 0.008; *post hoc* test: baseline vs. DynA: *p* = 0.019; baseline vs. wash: *p* = 1; wash vs. DynA: *p* = 0.019; *n* = 11; Figures 4C,D). The failure rate of eIPSCs increased from 11.5 ± 2.7% during baseline conditions to 43.2 ± 9.2% in presence of DynA and reversed to 12.5 ± 4.2% after wash out of DynA (One-Way ANOVA: *F*_(2,29)_ = 8.67; *p* = 0.001; *post hoc* test: baseline vs. DynA: *p* = 0.003; baseline vs. wash: *p* = 1; wash vs. DynA: *p* = 0.005; Figure 4D). Neither the mean latency of the eIPSCs (baseline: 2.2 ± 0.2 ms; DynA: 2.3 ± 0.2 ms), nor the eIPSC kinetics such as rise time_10–90%_ or decay time constant τ were significantly altered by DynA (rise time baseline: 1.2 ± 0.1 ms, DynA: 1.4 ± 0.2 ms; decay time constant baseline: 13.3 ± 0.7 ms, DynA: 14.6 ± 0.9 ms; Figure 4E). These data provide evidence that DynA negatively affects the efficacy of evoked GABAergic synaptic transmission onto NPS neurons.

**Figure 5:**
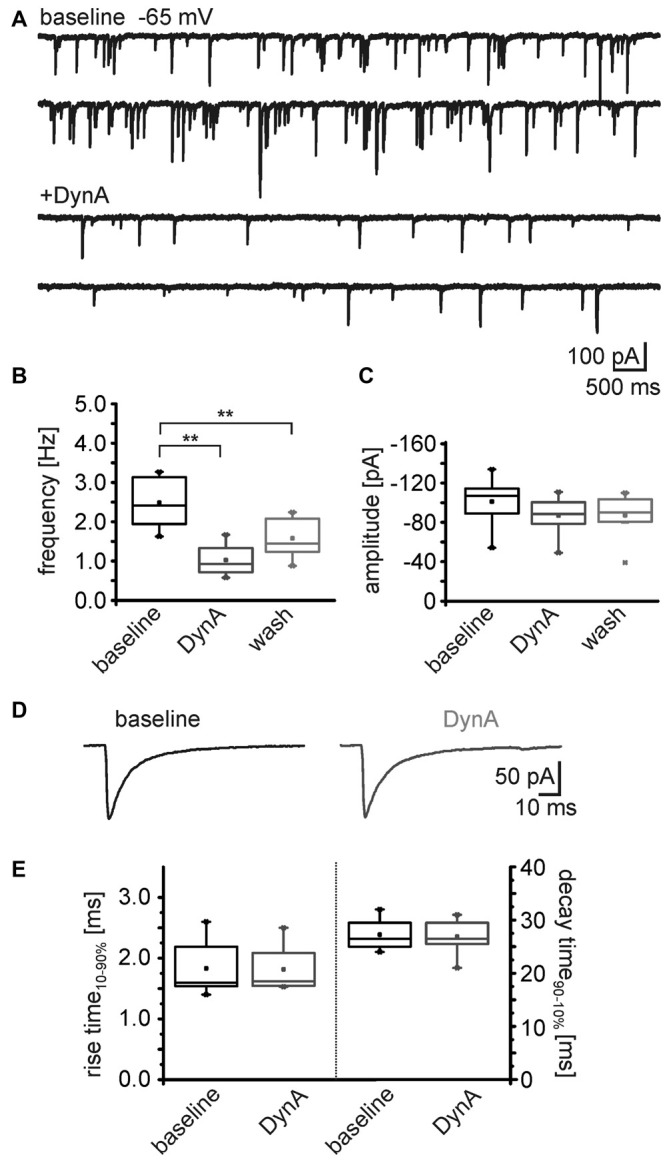
**DynA reduces the frequency of miniature inhibitory postsynaptic currents (mIPSCs) in NPS neurons. (A)** Sample voltage-clamp recording of mIPSCs in an NPS neuron at a holding potential of −65 mV during baseline conditions and in presence of DynA. Quantification of the normalized frequency **(B)** and normalized amplitudes **(C)** of mIPSCs during baseline, in presence of DynA and after wash (*n* = 10). **(D)** Examples of mean mIPSCs recorded during baseline conditions and in presence of DynA. **(E)** Application of DynA affects neither rise time nor decay time constants of mIPSCs.

To identify the possible site of action (pre- or postsynaptic) of the dynorphin system on GABAergic transmission, mIPSC in NPS neurons were recorded in voltage-clamp mode at a holding-potential of –65 mV and in presence of 0.5 μm TTX (Figure 5A). The mIPSC frequency was 2.5 ± 0.2 Hz (*n* = 8) during baseline recordings. The application of 250 nM DynA induced a reduction in mIPSC frequency to 1.0 ± 0.2 Hz, which reversed to 1.6 ± 0.2 after wash (One-Way ANOVA: *F*_(2,21)_ = 15.8; *p* = 6.485E-5; *post hoc* test: baseline vs. DynA: *p* = 4.753E-5; baseline vs. wash: *p* = 0.0071; wash vs. DynA: *p* = 0.1395; *n* = 8; Figures 5A,B). The mean amplitude of the mIPSCs during baseline recordings was −101 ± 8.8 pA. Application of DynA slightly reduced the amplitude to −87 ± 6.8 pA, which remained at 87.1 ± 8 pA after wash (One-Way ANOVA: *F*_(2,21)_ = 1.06; *p* = 0.364; Figure 5C). DynA did not affect the mIPSC kinetics (rise time_10–90%_ and decay time_90–10%_), which were extracted from thirty averaged events during baseline and DynA application (Figure 5D). The rise time was 1.8 ± 0.2 ms under control conditions and 1.8 ± 0.2 ms in presence of DynA (*p* = 0.71; *t* = 0.394; DoF = 7; *n* = 8), and the decay time_90–10%_ was 27.3 ± 1.0 ms under control conditions and 26.9 ± 1.2 ms in presence of DynA (*p* = 0.55; *t* = 0.63; DoF = 7; *n* = 8; Figure 5E). The fact that DynA mainly affected the frequency of mIPSCs points to an involvement of presynaptic mechanisms.

### Differential Distribution of KOR1 and KOR2 Receptors

**Figure 6:**
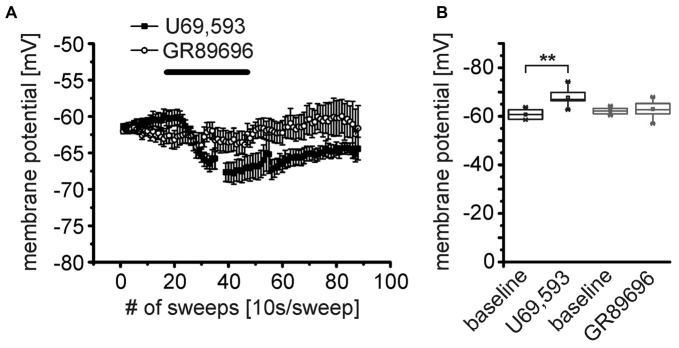
**The κ-opioid receptor 1 (KOR1) agonist U69,593 mimics the effect of DynA on the membrane potential of NPS neurons. (A)** Effect of the KOR1 agonist U69,593 (250 nM; *n* = 7) and the KOR2 agonist GR89696 (250 nM; *n* = 12) on the membrane potential of NPS neurons. **(B)** Quantification of the effects of KOR1 and KOR2 agonists on the membrane potential. Note the lack of effect of the KOR2 agonist GR89696.

At least two KOR subtypes, KOR1 and KOR2, can be differentiated pharmacologically (Zukin et al., 1988). Here, we used the KOR1 agonist U69,593 and the KOR2 agonist GR89696 (both at a concentration of 250 nM) to pharmacologically analyze the functional expression profile of these two receptor subtypes pre- and postsynaptically. During recordings of the membrane potential in presence of TTX, DNQX, CGP55845, GBZ and AP-5, U69,593 induced a significant hyperpolarization of NPS neurons (Figure 6A). The NPS neurons hyperpolarized from −61 ± 1 mV during baseline recordings to −67.4 ± 0.6 mV (*p* = 9.815E-4; *t* = 5.98; DoF = 6 *n* = 7; Figure 6A). In contrast, GR89696 failed to induce a significant hyperpolarization (baseline: −62.1 ± 0.4 mV; GR89696: −63.0 ± 1.1 mV; *p* = 0.33; *t* = 1.02; DoF = 11; *n* = 12; Figure 6A). The maximal shift of the membrane potential (ΔV_membrane_) was significantly larger upon U69,593 application (−7.0 ± 1.2 mV) compared to the ΔV_membrane_ induced by GR89696 (−1.0 ± 0.9 mV; *p* = 2.005E-4; *t* = −4.71; DoF = 17; Figure 6B). These data indicate that NPS neurons express KOR1, but not KOR2 postsynaptically.

**Figure 7:**
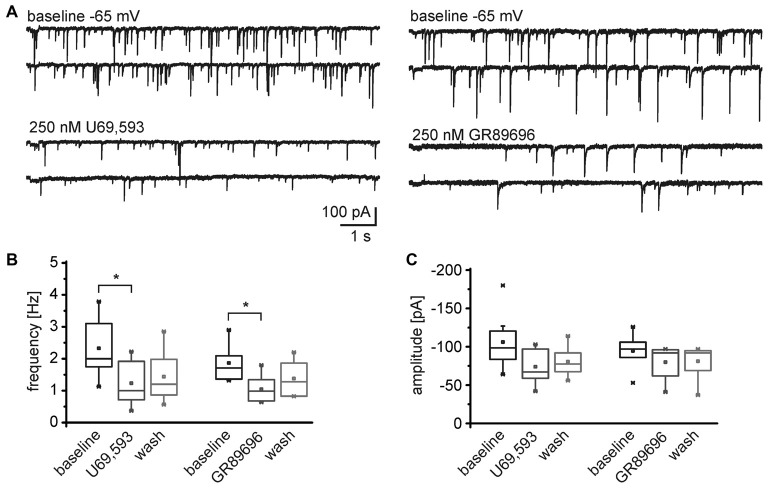
**Both, KOR1 and KOR2 agonists, reduce the frequency of mIPSCs in NPS neurons. (A)** Examples of mIPSCs recorded during baseline conditions and in presence of U69,593 (left) or GR89696 (right) in the voltage-clamp mode at a holding potential of −65 mV. Quantification of the normalized frequency **(B)** and normalized amplitudes **(C)** during baseline conditions, in presence of U69,593 (*n* = 7) or GR89696 (*n* = 7), and after wash.

Conversely, both, the KOR1 and KOR2 agonists, modulated the frequency of mIPSCs recorded from NPS neurons in the periLC region (Figure 7A). The KOR1 agonist U69,593 reduced the mIPSC frequency from 2.3 ± 0.3 Hz during baseline recordings to 1.2 ± 0.3 Hz (1.4 ± 0.3 Hz after wash; One-Way ANOVA: *F*_(2,21)_ = 4.14; *p* = 0.031; *post hoc* test: baseline vs. U69,593: *p* = 0.0397; baseline vs. wash: *p* = 0.118; wash vs. U69,593: *p* = 1; *n* = 8; Figure 7B). The mIPSC amplitude decreased from −106 ± 13 pA to −74 ± 8 pA in presence of U69,593 (−81 ± 7 pA after wash; One-Way ANOVA: *F*_(2,21)_ = 3.28; *p* = 0.058; Figure 7C). Similarly, the KOR2 agonist GR89696 induced a decrease in frequency from 1.9 ± 0.2 Hz during baseline to 1.1 ± 0.2 Hz (1.3 ± 0.2 Hz after wash; One-Way ANOVA: *F*_(2,18)_ = 4.84; *p* = 0.021; *post hoc* test: baseline vs. GR89696: *p* = 0.019; baseline vs. wash: *p* = 0.25; wash vs. GR89696: *p* = 0.68; *n* = 7; Figure 7B). The amplitudes shifted from −115 ± 14 pA during baseline to −98 ± 13 pA and −102 ± 14 pA in presence of GR89696 and after wash, respectively (One-Way ANOVA: *F*_(2,18)_ = 0.95; *p* = 0.4; *n* = 7; Figure 7C). In summary, both, KOR1 and KOR2 agonists, affect the frequency of mIPSCs, indicating a presynaptic mode of action of both pharmacologically distinct receptor subtypes.

## Discussion

The NPS system has been shown to be anxiolytic (Xu et al., 2004; Jüngling et al., 2008; Meis et al., 2008) and to attenuate stress-induced impairment of fear extinction (Chauveau et al., 2012). NPS neurons in the periLC depolarize in response to CRF application and subsequent CRF1 receptor activation (Jüngling et al., 2012). Moreover, stress exposure increases the expression of the immediate early gene cfos in NPS neurons (Jüngling et al., 2012), and NPS levels are increased during stressful encounters in the basolateral amygdala *in vivo* (Ebner et al., 2011). These data strongly suggest that the NPS system is active during stressful experiences. Conversely, there is evidence that a variety of stress effects, such as aversion and depressive-like effects are mediated by the kappa opioid system (Mague et al., 2003; McLaughlin et al., 2003; Smith et al., 2012; Van Bockstaele et al., 2010). Additionally, interference with the kappa opioid system by KOR antagonists reduces anxiety-like behavior (Knoll et al., 2007, 2011; Wittmann et al., 2009).

While the gene expression profile of NPS neurons in the periLC indicates the presence of KORs as well as DORs and MORs (Liu et al., 2011), only the endogenous KOR ligand DynA but neither a specific MOR nor a specific DOR agonist induced a strong hyperpolarization of the membrane potential of NPS neurons. Using membrane potential as read out of receptor stimulation, these data indicate that, in contrast to KORs, MORs and DORs are not expressed postsynaptically in the NPS neurons. This conclusion does not exclude the possibility that MORs and DORs are functionally expressed at presynaptic sites.

The data presented here provide evidence that NPS neurons are inhibited following KOR activation. Conversely, NPS neurons are activated by the activation of CRF1 by either CRF or stressin I (Jüngling et al., 2012). Since DynA and CRF are co-expressed e.g., in LC projecting neurons of the central amygdala (Reyes et al., 2011), both substances might be co-released. Co-application of DynA and CRF induced a biphasic response in NPS neurons, displaying a hyperpolarization followed by a depolarization upon wash. These data indicate that DynA is apt to attenuate CRF-induced activation of NPS neurons. Contrariwise, CRF diminishes the DynA-dependent hyperpolarization.

Application of DynA decreased the excitability and the input resistance of NPS neurons. Both effects seem to be mediated by an increase in the potassium conductance. It has previously been shown that KORs are intracellularly linked to inhibitory G_i/o_-proteins (Law et al., 2000), and activate inwardly rectifying potassium channels (GIRK; Henry et al., 1995). The DynA-induced outward current displayed rectifying properties and was sensitive to Cs^+^ in the internal solution, strongly arguing for an activation of potassium conductances in NPS neurons in response to DynA. The inclusion of cAMP in the intracellular solution did not significantly alter the DynA-induced current, excluding a potential role of adenylyl cyclases which has been observed in other types of neurons (Attali et al., 1989; Lawrence and Bidlack, 1993). Moreover, in presence of barium, the DynA-induced current was abolished, arguing for a major contribution of inwardly rectifying potassium channels. Of the four G protein-coupled inwardly rectifying potassium channel subunits (GIRK1–4) expressed in the rodent brain (Kobayashi et al., 1995; Karschin et al., 1996), the subunits GIRK1 and GIRK4 are sensitive to tertiapin Q, whereas GIRK2 and GIRK3 are insensitive (Jin et al., 1999). The DynA-induced current in NPS neurons was significantly reduced in presence of tertiapin Q, indicating the activation of GIRK1/4 downstream of the KOR. As seen in the box plots (Figure 3F), the DynA-induced currents were not abolished in all recorded neurons, pointing to the expression of tertiapin Q-insensitive GIRKs. In addition, G protein signaling beyond the canonical seven transmembrane domain receptors and G protein-independent pathways of these receptors may exist, including roles in receptor tyrosine kinase signaling and activity of G-protein accessory proteins (Sun et al., 2007; Coulon et al., 2010; Marty and Ye, 2010; Sato and Ishikawa, 2010).

Pharmacologically, two KORs, KOR1 and KOR2, can be distinguished by the use of specific agonists (Nock et al., 1988; Zukin et al., 1988; Rothman et al., 1989; Butelman et al., 2001). The KOR1-specific agonist U69,593, but not the KOR2 agonist GR89696, mimicked the hyperpolarizing effect of DynA in NPS neurons. A similar differential effect of KOR1 and KOR2 receptors has been described in proopiomelanocortin neurons (POMC) in the arcuate nucleus where KOR2 agonists but not KOR1 agonists induce a GIRK-dependent outward current (Zhang and van den Pol, 2013). Taken together, DynA induces a hyperpolarization and decreases the excitability of NPS neurons via KOR1.

The kappa opioid system has been shown to negatively regulate the release of GABA from synapses in various neuronal circuits (Hjelmstad and Fields, 2001, 2003; Li et al., 2012). During local electrical stimulation of GABAergic inputs onto NPS neurons in the periLC, application of DynA induced a significant increase in the failure rate of the evoked release. Concomitantly, a clear trend towards a transiently decreased mean amplitude of evoked IPSCs was observed. In contrast, the kinetic of eIPSCs was largely unaffected. Moreover, DynA and the KOR1- and KOR2-specific agonists, U69,593 and GR89696, respectively, reduced the frequency of GABAergic mIPSCs recorded in the presence of TTX. These data indicate that KORs can reduce GABA release by a presynaptic mode of action. From our data it cannot be concluded whether KOR1 and KOR2 receptors are expressed in the same or in separated synaptic terminals. It is known that there is formation of heterodimers of G protein-coupled receptors, which affects receptor pharmacology and signaling of the interacting subtypes (Prinster et al., 2005). Whether or not such an interaction of KOR1 and KOR2 plays a role in GABAergic afferents to NPS neurons in the periLC should be investigated in future studies.

It has previously been shown that the KOR1 agonist U69,593 attenuates GABA release by a reduction of the presynaptic Ca^2+^ influx via N-type channels (Hjelmstad and Fields, 2003). Effects of DynA on the frequency of GABAergic IPSCs have also been described for POMC and neuropeptide Y neurons in the arcuate nucleus (Zhang and van den Pol, 2013). Thus, it seems that mainly presynaptic mechanisms contribute to the observed reduction of the GABAergic synaptic transmission by KOR agonists. This would imply that KOR1 and KOR2 are present at presynaptic terminals of GABAergic synapses, whereas only KOR1 mediates the observed hyperpolarization via a direct postsynaptic mechanism. The activation of either presynaptic or postsynaptic located KORs could have differential effects on the excitability of NPS neurons. Activation of somatic KORs, inducing a prominent hyperpolarization, most likely would inactivate NPS neurons, and subsequently would cease the release of glutamate and NPS in the target regions of these neurons. Conversely, a predominant KOR activation on GABAergic terminals could mediate a disinhibition of NPS neurons due to a reduction of GABA release. Dynorphin could also act in an autoinhibitory mode since GABAergic afferents to NPS neurons in the periLC can contain dynorphin (Jüngling et al., 2015). In this scenario, heavily active GABAergic terminals control the amount of GABA release by co-releasing dynorphin. The net effect of KOR activation, either pre- and/or postsynaptically, on NPS neurons in the periLC *in vivo* remains elusive and demands further investigation.

Dynorphin-containing afferents can arise from various brain regions such as the hypothalamic paraventricular nucleus (PVN; Watson et al., 1983; Reyes et al., 2005), the bed nucleus of the stria terminalis (BNST; Van Bockstaele et al., 1999b), the nucleus of the solitary tract (NTS; Van Bockstaele et al., 1999a) or the central nucleus of the amygdala (CeA; Reyes et al., 2008, 2011). These brain regions send afferents to neurons of the LC and are involved in stress responses. Recently, neurons of the CeA expressing prodynorphin and/or somatostatin have been shown to form GABAergic synapses onto NPS neurons in the periLC (Jüngling et al., 2015), bringing them into the ideal position to counteract the activity of NPS neurons during stress exposure or anxiety. Based on these findings, it is tempting to speculate that anxiolytic-like effects in rats upon KOR antagonism (Knoll et al., 2007) are influenced by a disinhibition of the NPS system and/or a more pronounced CRF effect on NPS neurons in absence of KOR activity. Since the NPS system is present in a variety of brain regions and modulates different cognitive and autonomic states, the interaction of the dynorphinergic and the NPS system can be manifold and demands further investigation. To date it is unclear during which conditions (relevant stressor or phasic vs. chronic stress) KORs in NPS neurons are activated. In conclusion, our data provide evidence indicating that the NPS and the kappa opioid system are interacting and that the endogenous opioid dynorphin is suited to inhibit NPS neurons at the LC. In turn, NPS release in putative target regions will decrease during opioid-mediated inactivation of NPS neurons, exerting a variety of physiological consequences such as increased anxiety or vulnerability to stress exposure.

## Author Contributions

KJ study design, performed experiments, writing of the manuscript, data analysis. PB: performed experiments, writing of the manuscript, data analysis. LG: performed experiments, writing of the manuscript. H-CP: study design, writing of the manuscript.

## Conflict of Interest Statement

The authors declare that the research was conducted in the absence of any commercial or financial relationships that could be construed as a potential conflict of interest.
